# Anxiety-Depressive Syndrome and Binge-Watching Among Young Adults

**DOI:** 10.3389/fpsyg.2021.689944

**Published:** 2021-07-16

**Authors:** Jolanta Starosta, Bernadetta Izydorczyk, Antoni Wontorczyk

**Affiliations:** Faculty of Management and Social Communication, Institute of Applied Psychology, Jagiellonian University, Kraków, Poland

**Keywords:** binge-watching, anxiety, depression, motivation, problematic binge-watching, behavioral addiction

## Abstract

In recent years, binge-watching becomes a highly popular way of spending free time. Even though binge-watching usually is related to entertainment, there are concerns about some negative and unhealthy outcomes of excessive form of this behavior. The study examined the predictive value of anxiety-depressive syndrome in explaining the symptoms of problematic binge-watching and the tendency to adopt a specific motivation to watch series. Research group consists of 645 Polish young adults. The State-Trait Anxiety Inventory, Depression Measurement Questionnaire, Viewing Motivation Scale, and Questionnaire of Excessive Binge-Watching were used in this study. The results of the path analysis show that anxiety-depressive syndrome and motivation to watch TV series are the significant factors in the manifestation of all symptoms of problematic binge-watching. Moreover, there is a significant relation between anxiety-depressive syndrome and motivation to watch TV series, which especially concerns escape motivation and motivation to deal with loneliness. Furthermore, motivation to deal with loneliness, escape motivation, and motivation to spend free time have mediating effect on the relationship between anxiety-depressive syndrome and problematic binge-watching. Results of this research show that there could be not only normative binge-watching behavior but also unhealthy and problematic form of this behavior.

## Introduction

In recent years, there have been numerous changes in the way media is consumed, an example of which is the development of streaming platforms and the growing popularity of binge-watching (Flayelle et al., 2020; Jay, 2021). According to the definition given by Netflix (2013) itself, binge-watching is a phenomenon of watching at least two episodes of a TV series during one session. The phenomenon was also defined in this way in this article. It is worth noting, however, that in the constantly expanding literature on the subject, there are many definitions of this phenomenon, which refer to both the number and length of watched episodes and the way they are consumed (Flayelle et al., 2020).

Due to the popularity of the case, the scientific literature increasingly disputes whether excessively intense binge-watching can be considered in terms of anti-health behavior with symptoms of behavioral addiction (Orosz et al., 2016; Trouleau et al., 2016; Flayelle et al., 2020; Starosta et al., 2020). Undoubtedly, binge-watching can be a positive way of spending free time. However, some studies indicate that there is a thin line between healthy and unhealthy binge-watching (Flayelle et al., 2020). Socioecological model of health indicates that social and cultural phenomenons contribute to the variability of individual’s health (Słońska, 1994). The development of streaming platforms led to growth of popularity of binge-watching and changed the way how people consume television (Boca, 2019). Similarly to other addictions to technologies, binge-watching could be perceived as a behavior which usually is not associated with high levels of social harm (Grzegorzewska and Cierpiałkowska, 2018; Ort et al., 2021). However, some studies indicate that excessive forms of binge-watching could be related to negative health and social consequences (Riddle et al., 2017; Flayelle et al., 2019b; Steins-Loeber et al., 2020). The newest studies search for the answer how healthy way of spending free time can change into the problematic binge-watching. The occurrence of anti-health form of binge-watching could be related to the new construction of narrative created by streaming platforms, characteristics of video on demand platforms, and individual psychological predispositions to development of problematic use of media (Alter, 2017; Grzegorzewska and Cierpiałkowska, 2018; Boca, 2019; Brand et al., 2019). Research has shown that binge-watching is an event that is very engaging both emotionally and cognitively, which may lead to loss of control over the number of watched episodes (Schweidel and Moe, 2016; Flayelle et al., 2017; Granow et al., 2018). The aforementioned loss of control is undoubtedly related to the production of series with a comprehensive narrative that keeps the viewer’s attention (Alter, 2017). It could also be related to the specificity of the streaming platforms themselves, which allow you to watch series without commercial breaks and automatically turn on the next episode of the series just after few seconds the previous episode has ended (Ahmed, 2017; Flayelle et al., 2019b). In addition, the easy availability of series on numerous devices – TV sets, desktops, tablets, or telephones as part of the wide range of streaming platforms (Trouleau, 2016; Sung et al., 2018) – may also play a role. Losing control over the amount of time spent on binge-watching may affect the entire functioning of an individual in the sphere of relations with other people, fulfilling duties, or taking care of health (Orosz et al., 2016; Chambliss et al., 2017; Exelmans and Van den Bulck, 2017; Riddle et al., 2017; Flayelle et al., 2019a, b). These issues are related to the criteria of Internet gaming disorder included in section III of DSM-5 by American Psychiatric Association (2013) and the criteria of gaming disorder distinguished by WHO (2018) in ICD-11. On this basis, it can be concluded that there are some similarities between addiction to new technologies and excessive binge-watching.

So far, there has been little research into the psychological determinants of excessive binge-watching. According to the researchers, the reasons for engaging in binge-watching behaviors, including those of a problematic nature, should be sought in personality traits, ways of regulating affect, and the motivation manifested by the individual (Flayelle et al., 2019b, 2020; Starosta and Izydorczyk, 2020). The undertaken research focused on the impact of the anxiety-depressive syndrome on binge-watching behavior and the mediating influence of the manifested motivation to watch TV series. There are many studies of fundamental behavioral addictions that link the problematic use with anxiety and depression (Mehroof and Griffiths, 2010; Van Rooij et al., 2014; Liu et al., 2018). The problematic use of social media or video games, as well as psychoactive substances, can be used to regulate affect by anxious and depressive persons in order to obtain positive gratification and protect themselves from negative affect (Cheetham et al., 2010; Nikmanesh et al., 2014). The same may be the case with problematic binge-watching behaviors (Flayelle et al., 2019a). Research conducted by Wheeler (2015) and Ahmed (2017) showed that there is a positive relation between the higher frequency of binge-watching behaviors and depression and a sense of loneliness. The existence of a similar relation is also indicated by studies by Sun and Chang (2021). In turn, Steins-Loeber et al. (2020) indicated that depressive symptoms are a clear predictor of losing control over binge-watching. On the other hand, the results obtained by Tefertiller and Maxwell (2018) showed no relation between depression and loneliness and binge-watching. As can be seen, there are not many studies on the relationship between anxiety and depression and the tendency to manifest excessive binge-watching behaviors. On the other hand, those that are known contain contradictory results. In connection with the above, it is worth investigating the role of anxiety-depressive factors in the manifestation of problematic binge-watching.

The factor involved in regulating affect is the individual’s motivation to watch TV series. Research on motivation is most often based on the uses and gratifications theory (Rubin, 1983; Steiner and Xu, 2018). This theory states that people are using media, such as television or the Internet, to meet certain needs and achieve certain goals (Pittman and Sheehan, 2015). Viewers watch series for various reasons, the most common of which is entertainment, relaxation, and social motivation (Pittman and Sheehan, 2015; Panda and Pandey, 2017; Rubenking and Bracken, 2018). Individuals watch series to get or maintain a positive affect. Another motivation indicated by researchers is cognitive motivation – an individual watches series because of the desire to obtain information (Shim and Kim, 2018). On the other hand, the research also shows a very large role of motivation to cope with loneliness and escapism, which enables the individual to escape from problems and regulate negative emotions (Panda and Pandey, 2017; Rubenking and Bracken, 2018; Flayelle et al., 2019a, b). It is worth noting that the phenomenon of escapism into the virtual world and using it to regulate affect is a characteristic pattern of behavioral addictions (Kim et al., 2017; Sprong et al., 2019). Investigating the motivations accompanying binge-watching behaviors and their determinants is extremely important for understanding the essence of problematic/excessive binge-watching. It is also important to understand how depressive and anxious features affect the individual’s motivation to watch TV series.

The path model of behavioral addictions by Blaszczynski and Nower (2002) or I-PACE model of addiction by Brand et al. (2019) shows that there are multiple ways to develop behavioral dependency. These models indicate personality traits, and affective disorders could be psychological predisposition of engaging in excessive forms of some behaviors or substance addictions. Multiple research on the problematic use of technology showed that there is bidirectional relationship between anxiety, depression, and problematic use of Internet or social media (Mentozoni et al., 2011; Männikkö et al., 2015; Mérelle et al., 2017; Krossbakken et al., 2018). As was mentioned before, there are some studies which indicate similar results in case of binge-watching (Ahmed, 2017; Sun and Chang, 2021). However, due to the dearth in the literature on this subject, the further research is needed. In relation to path model (Blaszczynski and Nower, 2002) and I-PACE model (Brand et al., 2019), these psychological predispositions could affect individual’s needs and motivations to engage in excessive forms of binge-watching. Multiple studies show that specific motivation to binge-watching is usually defined on the basis of Uses and Gratification Theory. Achieving individual’s goal and gratifying their needs could enhance the behavior and lead to developing problematic binge-watching.

It seems important to conduct research on the role of anxiety, depression, and motivation for the manifestation of problematic binge-watching as the constant increase in the popularity of binge-watching behaviors and the indications appearing in the scientific literature about the possibility of the existence of excessive forms of the phenomenon, characterized by features similar to behavioral addictions, can be observed. The study of the relations between the variables is important to understand the determinants underlying excessive forms of binge-watching and thus to understand the characteristics underlying anti-health behaviors. Furthermore, understanding the mechanism of problematic binge-watching is significantly important in the population of young adults. The entering the adulthood is a period of life with high risk of development of substance and behavioral addictions (Sussman and Arnett, 2014; Lopez-Fernandez et al., 2017). Statistical research shows that young adults tend to binge-watch on daily basis, which makes them population with higher risk of developing tendency for excessive binge-watching (Sabin, 2018). The results may have an impact on the development of preventive and therapeutic measures toward people prone to problematic binge-watching. In addition, the results of the research will contribute to a better understanding of the phenomenon of binge-watching, which, although it was established in 2013, is still an unexplored phenomenon from a psychological perspective.

### Research Objective and Questions

According to path model of behavioral addictions (Blaszczynski and Nower, 2002) and I-PACE (Brand et al., 2019), there are psychological predispositions, such as personality, psychopathology, and tendency, to present special motivation and needs can lead to the development of behavioral addiction.

**Figure 1:**
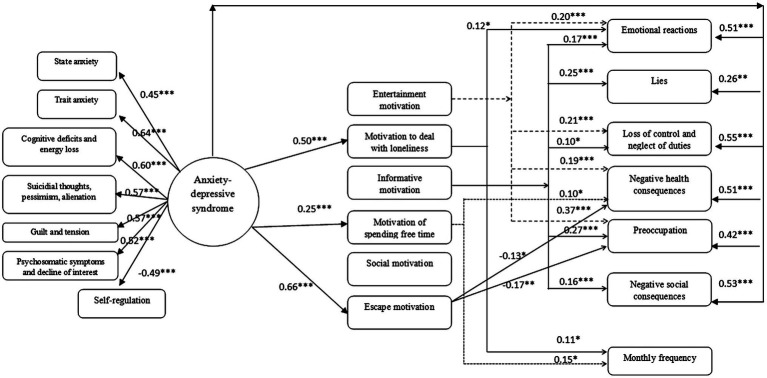
Theoretical model.

As you can see in the model presented in the Figure 1, the explanatory variable has been called the anxiety-depressive syndrome. According to the literature, anxiety and depressive traits are invariably correlated, because anxiety is both a characteristic symptom of anxiety and depressive disorders (Butcher et al., 2020). Often people suffering from anxiety disorders are also characterized by the coexistence of depressive disorders. The personality trait-neuroticism is also associated with anxiety and a tendency to experience negative, depressed mood, as well as a tendency to rumination. The anxiety-depressive syndrome was defined with reference to the structural model of Cicchetti et al. (1995) as a cognitive-affective structure related to the individual’s tendency to react with anxiety and depressive mood in various life situations. These dispositions affect the cognitive and emotional functioning of an individual, the motivation manifested by them, and their behavior. The anxiety components of the anxiety-depressive syndrome are based on the theory of Spielberger (1966), who distinguished anxiety-state and anxiety-trait. In turn, the depressive components refer to the International Classification of Diseases (ICD) and Diagnostic and Statistical Manual of Mental DIsorders (DSM) criteria for understanding depression.

The first component of the anxiety-depressive syndrome is state anxiety. A given factor is defined as a subjective and conscious feeling of tension accompanied by the stimulation of the autonomic nervous system at a specific point in time (Spielberger, 1966). State anxiety is thus understood as the state of tension currently experienced by the individual. The second component of the anxiety-depressive syndrome is trait anxiety, defined as a behavioral disposition to react with disproportionate high anxiety despite the lack of objective danger and perceiving harmless situations as causing anxiety. Another factor included in the anxiety-depressive syndrome is cognitive deficits and energy waste, which determine the subjective sense of difficulty of an individual in the functioning of his cognitive processes and the feeling of energy loss to act and carry out everyday activities. The fourth factor is thinking about death, pessimism, and alienation, which are defined as the subjective feelings of hopelessness, emotional emptiness, alienation, and isolation. The fifth factor was guilt and tension, which describes the level of intensity of negative emotions, such as sadness, guilt, being punished, and anxiety, and also measures the individual’s tendency to rumble. Another factor was Psychosomatic Symptoms and Loss of Interests, which were defined as the severity of one’s own health deficits–activities, such as sleep, wakefulness, movement, and sex. This variable also measures the level of decline in pleasure derived from various activities and interests. The last component is self-regulation, which is defined as the level of an individual’s cognitive-emotional resources to deal with negative emotions.

The variable explained is the problematic binge-watching, which was defined on the basis of the model of behavioral addiction proposed by Griffiths (2005), the criteria of gaming disorder (WHO, 2018), and Internet gaming disorder included in section III of DSM-5 (American Psychiatric Association, 2013). The criteria of abovementioned disorders focus on the impaired control over the individual’s behavior, preoccupation, tolerance, continuation, and escalation of the behavior despite negative consequences and using the behavior to relieve negative moods (Griffiths, 1996; American Psychiatric Association, 2013; WHO, 2018). On the basis of these criteria, problematic binge-watching is defined as persistent and excessive binge-watching, characterized by a loss of control over the manifested behavior in order to obtain gratification and escaping negative emotions, excessive cognitive-emotional preoccupation, and loss of other interests in favor of binge-watching. The behavior of an individual has an impact on the performance of duties and is associated with negative social and health consequences.

The problematic binge-watching consists of six components, also known as symptoms. The first one, called emotional reactions, is associated with defining binge-watching as a source of positive emotions, as well as a way to regulate negative emotional states. It also includes an emotional discomfort (feeling of anger, anxiety, and depression) in a situation in which the individual cannot binge-watch. The second component is lie, defined as the tendency of an individual to hide the truth about the amount of time spent on binge-watching. The third component is loss of control and neglect of duties, defined as the loss of control over the amount of time spent on binge-watching, which may result in the neglect of employee, family, or educational duties by an individual. The fourth component is negative health consequences. This variable measures the intensity of sleep problems and irregular and unhealthy diets that are a consequence of excessive binge-watching. The fifth symptom is preoccupation, and it is a variable expressing the cognitive and emotional fascination with binge-watching, manifested by involvement in binge-watching itself, as well as searching for additional information about series. The last component is negative social consequences, which express the intensity of the subjective assessment of the deterioration of relationships with other people as a result of the amount of time spent on binge-watching.

The next variables explained are binge-watching behaviors which consist the frequency of binge-watching sessions during a month and the number of watched episodes during one binge-watching session. These variables measure the intensity of undertaken behaviors and the number of watched episodes.

The motivation to watch TV series is the variable mediating the relation between the anxiety-depressive syndrome and problematic binge-watching, the frequency of sessions, and the number of episodes watched during one session. The definition of this variable was based on the uses and gratifications theory (Rubin, 1983; Steiner and Xu, 2018). This theory says that the use of media, such as television or computer, directs the behavior of an individual to meet their needs and achieve goals.

On the basis of Rubin’s (1983) motivation to watch TV series, the following six motivations were distinguished. Entertainment motivation is defined as watching series in order to relax and to have fun and arouse positive emotions. Motivation to deal with loneliness is defined as a behavior undertaken by an individual to avoid feeling lonely. The characters of the series become “companions” for the individual, thanks to which the individual lowers his sense of isolation and sadness caused by the lack of the company of other people. A different factor is informative motivation, defined as watching a series induced by the cognitive needs of an individual. The viewer watches the series to get information about the world, other people, and himself. Another type of motivation is motivation to spend free time, which describes watching TV series as a habitual activity aimed at counteracting the feeling of boredom. There is also social motivation – by watching serials, an individual initiates, maintains, and deepens relationships with other people. As a result, the person spends time with them, is part of the group, and can exchange opinions about the series with other people. The last component of the mediating variable is escape motivation, which characterizes individuals who use series to escape from the problems of the everyday world. That way individuals are able to distract themselves from negative feelings.

The aim of this article is to identify the predictive role of the anxiety-depressive syndrome in the tendency to manifest symptoms of problematic binge-watching and the tendency to adopt a specific motivation to watch series. Another goal of the article is to determine how the motivation to watch series influences the symptoms of problematic binge-watching. Furthermore, the goal of the study is to identify the mediating effect of motivation to watch TV series on relationship between the anxiety-depression syndrome and the symptoms of problematic binge-watching.

In connection with the above, the following research questions were asked:

1. Does anxiety-depressive syndrome explain the symptoms of problematic binge-watching among young adults and to what extent?
2. Does anxiety-depressive syndrome explain the occurrence of particular types of motivation to watch TV series among young adults and to what extent?
3. Do and to what extent the motivations to watch TV series mediate the relation between the anxiety-depressive syndrome and the symptoms of problematic binge-watching among young adults?
4. Does anxiety-depressive syndrome and motivation to watch TV series explain the binge-watching behaviors – monthly frequency of binge-watching session and the number of binge-watched episodes in one sitting?

Due to the exploratory character of this research, it was decided to not provide specific hypothesis. However, on the basis of the abovementioned studies, it can be assumed that the anxiety-depressive syndrome indirectly influences the symptoms of problematic binge-watching.

## Materials and Methods

### Methods

The Polish adaptation of the STAI – State-Trait Anxiety Inventory by Spielberg and Lushene (Wrześniewski and Sosnowski, 1996) – was used to study the anxiety-depressive syndrome. The inventory is a tool designed to measure the severity of state anxiety and trait anxiety. The inventory is characterized by satisfactory psychometric indicators. Internal reliability coefficient of the individual scales in Polish adaptation by Wrześniewski and Sosnowski (1996) is between 0.88 and 0.91. The Polish adaptation of the questionnaire consists of 40 items. People participating in the study are asked to rate their answers on a 4-point scale. The respondents were given the following types of statements to choose from depending on the scale tested: 1– “definitely not”/“almost never,” 2 – “rather not”/“sometimes,” 3 – “rather yes”/“often,” and 4 – “definitely yes”/“almost always.”

The second tool measuring the anxiety-depressive syndrome was the Depression Measurement Questionnaire by Łojek et al. (2015). The tool allows you to measure feelings, thoughts, and depressive behavior. It consists of the following five scales: cognitive deficits and energy loss; thinking about death, pessimism, and alienation; guilt and anxiety tension; psychosomatic symptoms and loss of interest; and self-regulation. The questionnaire also consists of an overall score, which is the sum of the first four scales (cognitive deficits and energy loss; thinking about death, pessimism, and alienation; guilt and anxiety; psychosomatic symptoms and loss of interest). This result determines the intensity of depressive symptoms in the examined person. The Depression Measurement Questionnaire has satisfactory psychometric indicators. The reliability coefficients ranged from 0.73 to 0.96 (Łojek et al., 2015). The questionnaire consists of 75 questions. The respondents are asked to rate their answers on a 4-point scale, where in the case of 66 items 1 – “always”/“constantly,” 2 – “often,” 3 – “sometimes,” and 4 – “never.” In the case of 9 items, the respondent chooses from the following answers: 1 – “very,” 2 – “significantly,” 3 – “slightly,” and 4 – “not at all.”

The Polish adaptation of Viewing Motivation Scale by Rubin was used to measure the level of the motivation for watching TV series (Rubin, 1983; Starosta, et al., 2019). The Polish adaptation of the questionnaire consists of 27 items and six scales: entertainment motivation, motivation to deal with loneliness, informative motivation, motivation of spending free time, social motivation, and escape motivation. The Cronbach’s *α* coefficients for the abovementioned scales ranged from 0.69 to 0.88 in Polish adaptation. It indicates satisfactory reliability of the tool. The intercorrelations between the constructs ranged between 0.09 and 0.43 (*p* < 0.001). Respondents rate their answer on a 5-point Likert scale, where 1 means – “completely untrue,” 2 – “a bit true,” 3 –“very likely,” 4 –“true,” and 5 – “definitely true.”

Another method used in this study was the Questionnaire of Excessive Binge-Watching created by Starosta et al. (2019). The questionnaire was used to examine the symptoms of problematic binge-watching, which can be the symptoms of the behavioral addiction. The authors of the tool conducted Exploratory Factor Analysis (EFA) which enables to distinguish 6 subscales from 30 items: emotional reactions, lies, loss of control and neglect of duties, negative health consequences, preoccupation, and negative social consequences. The Cronbach’s α coefficients calculated on the basis of Polish population for the whole method and separate subscales of the questionnaire ranged from 0.67 to 0.89 which indicate satisfactory psychometric characteristic of the tool. The intercorrelations between the constructs ranged between 0.26 and 0.61 (*p* < 0.001). Participants of the study mark their answers on the 6-point Likert scale: 1 – “never,” 2 – “sporadically,” 3 – “rarely,” 4 – “sometimes,” 5 – “often,” and 6 – “always.” The Questionnaire of Excessive Binge-Watching can also be used to measure the level of intensity of problematic binge-watching among the Polish students. The total score of this method determines whether risk of occurring symptoms of excessive binge-watching is low (0–60), medium (61–120), or high (121–180).

### Data Analytic Procedures

Firstly, descriptive statistics were measured in terms of the intensity of all variables and their indicators included in the research model. Secondly, the research model was tested by using path analysis – analysis of structural equations. The results were obtained by using AMOS-SPSS (Arbuckle, 2014) software. Due to the improvement of readability of the result, the path analysis model consists only significant paths. The obtained results of the analyses are presented in a section results.

### Characteristics of the Research Group

The study was conducted from September 2020 to January 2021. The research was carried out in the form of an individual study online using the Microsoft Teams platform. The study was voluntary, and the respondents consented to participate. The study was anonymous, and no personal data were collected during the study. Selection for the research group was deliberate. The criteria for inclusion in the group were declaring watching TV series – watching two or more episodes of TV series in one sitting, being between the ages of 18 and 30 declaring no information about the occurrence of diagnosed mental illness, such as affective, psychotic, or anxiety disorders, substance dependence, and occurrence of treatment – psychotherapy and/or pharmacotherapy – of abovementioned disorders. The exclusion criteria were related to not watching TV series or watching less than two episodes of TV series, being under the age of 18 or over 30 and having diagnosed mental illness (affective, psychotic, and anxiety disorders, substance dependence, occurrence of mental illness treatment – psychotherapy and pharmacotherapy). A study of 600 people was planned. In developing the research model, the authors estimated the minimum size of the research sample (*n* = 384). Initially, the research sample consisted of 752 people, but due to the failure to meet the inclusion criteria and the deficiencies in the supplement to the questionnaire, 107 people were removed from the research sample.

The research group consisted of 645 people. The study included 537 women (83.26%), 98 men (15.19%), and 3 transgender men (0.47%). It is worth mentioning that seven people decided not to give their gender (1.09%). The average age of the respondents was 20 years. The half of the respondents are people who only study (54.11%). In turn, people who study and work at the same time constitute 32.40% of the studied group. On the other hand, working people constitute 13.49% of all respondents. The percentage distribution of individual fields of study among the respondents is as follows: social sciences – 49.92%, humanities – 28.68, exact sciences – 6.36%, and medical sciences – 3.88%. People who did not study accounted for 10.23% of the research group. Another characteristic of the research group was their relationship status. This variable divides the group in half because at the time of the study, 49.30% of the respondents were single, while 50.70% were in a relationship.

### Characteristic of Binge-Watching Among Polish Young Adults

The next stage of the research was to collect the characteristics related to binge-watching. The vast majority of respondents admit that binge-watching happens (95.66%). The analysis of the respondents’ responses indicates that most of the respondents watch from two to five episodes during one binge-watching session (81.55%). It should be noted, however, that the remaining group of respondents – as many as 18.45% – indicated that they watch from 6 to 20 episodes during one binge-watching session. In terms of the number of binge-watching sessions, the majority of respondents indicated that binge-watching was performed 1–5 times a month (66.82%), while the remaining respondents (33.18%) admitted that they sometimes binge-watch 6–30 times a month. In terms of the preference of where to watch series, all respondents replied that they perform binge-watching at home. Only 9.61% of respondents admitted that they binge-watch while traveling, and 2.33% answered that they sometimes binge-watch at work. The vast majority of respondents prefer to binge-watch alone (83.72%). Only 30.52% of respondents answered that they sometimes watch TV series with a partner, 9.92% indicated that binge-watching is carried out by the whole family, and only 6.98 replied that they were watching series with friends. The most common binge-watching genres among the respondents were comedies, crime stories, action/sensational series, sci-fi/fantasy series, and drama series.

## Results

In the first stage of the statistical analysis of the obtained results, descriptive statistics were measured in terms of the intensity of all variables and their indicators included in the research model. The obtained results are presented in Table 1.

**Table 1:** Descriptive statistics for the intensity of researched variables.

Variables	*n*	*M*	Me	Min	Max	*SD*
State anxiety	645	41.76	41.00	21.00	77.00	11.087
Trait anxiety	645	46.47	46.00	20.00	76.00	10.615
CDEL	645	42.23	41.00	19.00	74.00	11.228
STPA	645	28.18	26.00	15.00	60.00	9.825
GT	645	35.58	35.00	16.00	63.00	9.749
PSDI	645	20.85	20.00	10.00	40.00	5.716
S	645	40.84	41.00	21.00	58.00	6.812
ODSS	645	127.68	122.00	64.00	233.00	33.268
EM	645	34.63	35.00	9.00	45.00	7.308
MDWL	645	7.32	7.00	3.00	15.00	3.471
IM	645	12.04	11.00	5.00	25.00	4.546
MSFT	645	13.01	13.00	4.00	28.00	4.271
MS	645	5.14	5.00	2.00	10.00	2.550
EM	645	9.03	9.00	3.00	15.00	3.376
RE	645	17.33	16.00	8.00	48.00	7.380
L	645	5.23	4.00	3.00	15.00	2.568
LCND	645	17.90	17.00	7.00	37.00	6.647
NHC	645	11.17	10.00	5.00	27.00	4.067
P	645	11.49	11.00	4.00	24.00	3.772
NSC	645	5.17	4.00	3.00	18.00	2.645
GBW	645	68.28	66.00	30.00	157.00	21.75
Frequency of BW session in a month	645	5.26	3.00	1.00	30.00	5.48
The number of episodes in one BW session	645	4.18	4.00	2.00	20.00	2.01

*CDEL, cognitive deficits and energy loss; STPA, suicidal thoughts, pessimism, and alienation; GT, guilt and tension; PSDI, psychosomatic symptoms and decline in interest; S, self-regulation; ODSS, overall depression severity score; EM, entertainment motivation; MDWL, motivation to deal with loneliness; IM, information motivation; MSFT, motivation to spend free time; ESM, escape motivation; RE, emotional reactions; L, lie; LCND, loss of control and neglect of duties; NCHC, negative health consequences; P, preoccupation; NSC, negative social consequences; GBW, general result of excessive binge-watching behavior; and BW, binge-watching*.

Based on the obtained descriptive statistics, it can be concluded that the research group is characterized by a moderate level of anxiety – both trait and state, and a moderate intensity of depressive traits. The participants of the study show an average intensity of the distinguished motivations to watch the series. Most of the respondents are characterized by an average intensity of symptoms of problematic binge-watching. People tested on average five times a month binge-watching. The average number of episodes watched during one binge-watching session is four.

**Figure 2:**
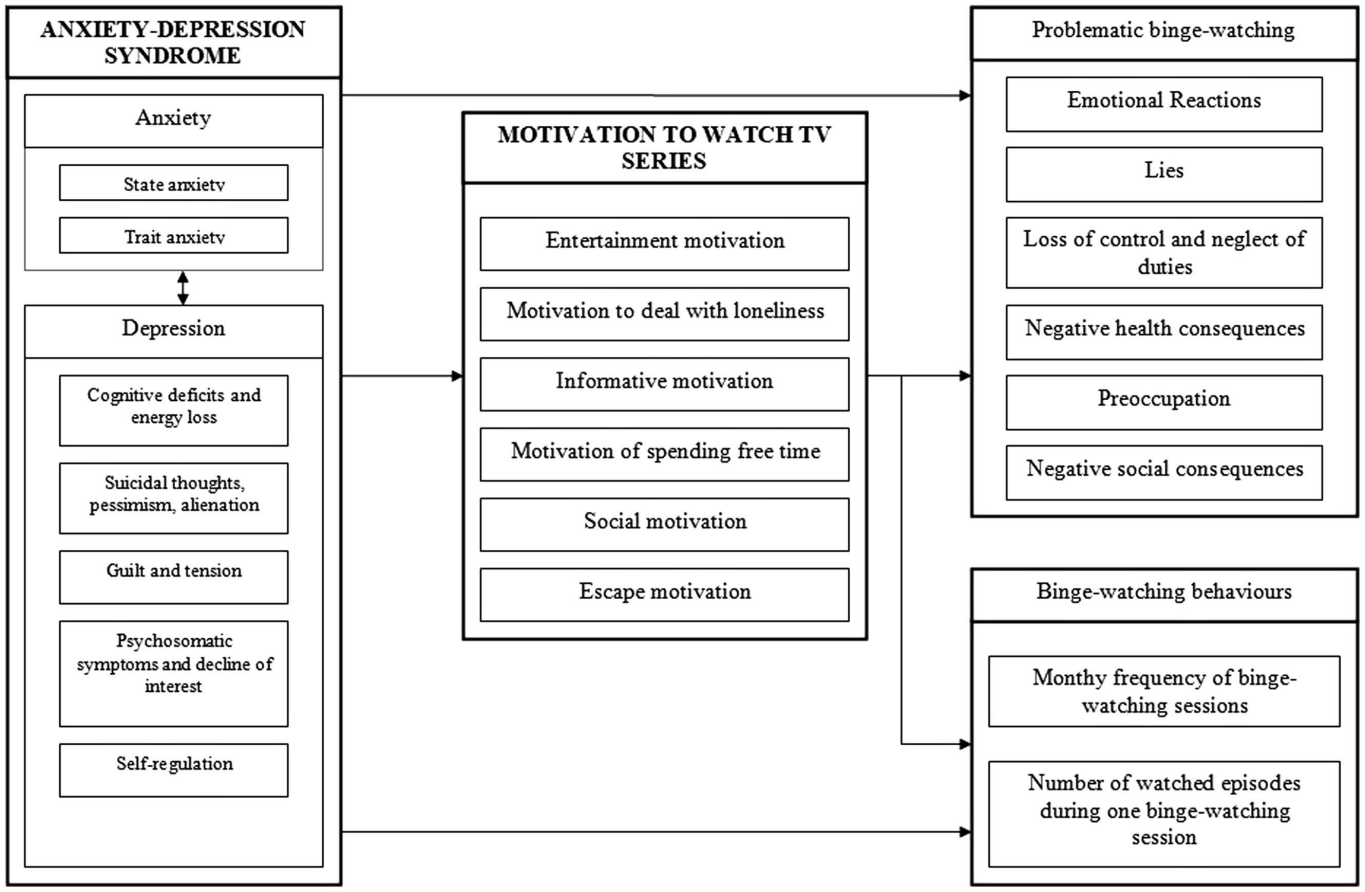
Path analysis model. ^*^p ≤ 0.05; ^**^p ≤ 0.01; ^***^p ≤ 0.001.

The next stage of the research was conducting the path analysis. To make the presentation of the results clear, only the relevant paths are presented in Figure 2. The variables distribution is normal. Due to the amount of method used in the study which were described on various point scales, variables were standardized to perform the path analysis. The standardized *β* coefficients ranged from −1 to 1. The chi-squared coefficient (Chi = 17.84; *df* = 72, *p* < 0.05) indicates that there is no significant differences between research model and obtained results of the analysis. Furthermore, the rest of the obtained indicators of the goodness of fit test, such as GFI = 0.976, AGFI = 0.929, and CFI = 0.950, allow the conclusion that this model is well represented by the correlation matrix which will be based on the collected empirical data.

Based on the results presented above, it can be concluded that trait anxiety, cognitive deficits and energy loss, suicidal thoughts, pessimism, and alienation, and guilt and anxiety tension are the variables which load the anxiety-depressive syndrome most significantly.

The obtained results of the analysis show that the anxiety-depressive syndrome has a direct and significant effect on all variables that are symptoms of problematic binge-watching. Anxiety-depressive syndrome is the strongest predictor of such variables as: loss of control and neglect of duties (*β* = 0.55^***^), negative social consequences (*β* = 0.53^***^), emotional reactions (*β* = 0.51^***^), and negative health consequences (*β* = 0.51^***^). The *β* coefficient for the variable preoccupation (*β* = 0.42^***^) indicates that the predictive value of anxiety-depressive syndrome is medium. Furthermore, this syndrome has also medium predictive value for the variable lies (*β* = 0.26^**^). The greater the intensity of the depression-anxiety syndrome, the greater the intensity of the symptoms of excessive binge-watching will be. The obtained analysis did not show that the anxiety-depressive syndrome was a significant predictor of the frequency of binge-watching sessions per month or the number of episodes watched during one binge-watching session.

The anxiety-depressive syndrome also influences the motivation shown by an individual to watch TV series. The highest *β* values were recorded in the case of escape motivation (*β* = 0.66^***^). This will mean that the greater the intensity of the anxiety-depressive syndrome, the greater the escape motivation to watch TV series. The anxiety-depressive syndrome is also a significant and strong predictor of motivation to deal with loneliness (*β* = 0.50^***^) and medium for the motivation to spend free time (*β* = 0.25^***^). The anxiety-depressive syndrome has positive effect on the all abovementioned variables. This mean that the greater the intensity of the anxiety-depressive syndrome, the higher the intensity of motivation to deal with loneliness and spending free time displayed by the respondents. The analysis shows that there is no significant relation between anxiety-depressive syndrome and such motivations as entertainment, informative, and social motivation to watch TV series.

In addition, the analysis of the matrix of total effect showed that the motivation to watch TV series has a weak mediating effect on the relationship between anxiety-depressive syndrome, symptoms of problematic binge-watching, and the frequency of binge-watching sessions within 1 month. The direct impact of anxiety-depression syndrome is medium and strong (*β* ranged from 0.26^**^ to 0.55^***^). The indirect influence of anxiety-depressive syndrome is weak. The obtained *β* coefficients ranged from −0.098^*^ to 0.080^*^, and most of them had negative impact which leads to the assumption that mediating effect of motivation may reduce the influence of anxiety-depressive syndrome on the problematic binge-watching. Due to that, it can be assumed that some motivation can weaken the effect of anxiety-depressive syndrome on problematic binge-watching.

The results of path analysis show that motivation to deal with loneliness, motivation to spend free time, and escape motivation mediate between anxiety-depression syndrome and symptoms of problematic binge-watching. Motivation to deal with loneliness had weak indirect impact on the emotional reactions (*β* = 0.12^*^). Another variable indirectly influencing the symptoms of problematic binge-watching is escape motivation, which negatively affects preoccupation (*β* = −0.17^**^) and negative health consequences (*β* = −0.13^*^). A person with high anxiety-depressive syndrome while manifesting escape motivation when watching TV series may have lower preoccupation and lower health consequences, such as insomnia and eating unhealthy food. The last motivation that has an indirect effect on the problematic binge-watching is the motivation to spend free time, which has the least impact on the negative health consequences variable (*β* = 0.09^*^).

Although no significant direct effect of the anxiety-depressive syndrome on the frequency of binge-watching sessions during 1 month was observed, the results show that it has weak indirect impact through the motivation to spend free time (*β* = 0.15^*^) and motivation to deal with loneliness (*β* = 0.11^*^). This means that people with high levels of anxiety-depressive syndrome who watch TV series to cope with loneliness or do it out of habit tend to binge-watch more frequently.

The entertainment motivation significantly and directly influences such symptoms of problematic binge-watching as preoccupation (*β* = 0.37^***^), loss of control and neglect of duties (*β* = 0.21^***^), emotional reactions (*β* = 0.20^***^), and negative health consequences (*β* = 0.19^***^). This means that the more an individual is motivated by entertainment, the greater their cognitive and emotional preoccupation with a TV series binge-watching, and they experience a greater intensity of positive emotions when watching a series and negative emotions when binge-watching is not possible. In addition, people with entertainment motivation are more likely to lose control over the amount of time they spend watching TV series, which may result in neglecting their duties or their health. Another variable which has direct effect on symptoms of problematic binge-watching is informative motivation. The informative motivation has the medium impact on the preoccupation (*β* = 0.27^***^) and lies (*β* = 0.25^***^). The relationship between this motivation and such variables as emotional reactions (*β* = 0.17^***^), negative social consequences (*β* = 0.16^***^), and loss of control and neglect of duties (*β* = 0.10^*^) was significant but weak.

The results show that social motivation does not affect the symptoms of problematic binge-watching or the frequency of binge-watching sessions during the month.

Although the theoretical model takes into account the number of episodes watched during one binge-watching session, the obtained results show no impact of the anxiety-depressive syndrome or motivational variables on this variable.

## Discussion

The results of the analysis show that there is a significant direct impact of the anxiety-depressive syndrome on the symptoms of problematic binge-watching. It can therefore be concluded that the stronger the intensity of anxiety-depressive symptoms, the greater the intensity of symptoms of problematic binge-watching. The research therefore confirms the results obtained by Wheeler (2015), Ahmed (2017), and Sun and Chang (2021), which also highlighted the existence of a relationship between depression and excessive binge-watching behaviors. The anxiety-depressive syndrome was strong predictor of such symptoms of problematic binge-watching as emotional reactions, loss of control, and negative social and health consequences. Perhaps, people with a higher intensity of anxiety and depression more often treat binge-watching as a source of positive affect. Individuals can consume a large number of episodes of a TV series in order to receive positive gratification and to escape from the negative emotional state they are in (Panda and Pandey, 2017; Rubenking and Bracken, 2018; Flayelle et al., 2019a, b). Moreover, emotional distress in the situation of not being able to watch TV series may exacerbate their basically depressed mood, which may induce individuals to take actions aimed at changing this state. The use of substances or excessive forms of behavior to regulate affective states is characteristic of all addictions (Nikmanesh et al., 2014; Mascia et al., 2020). Research has shown that in the case of behavioral addictions, the most common symptoms of abstinence are emotional tension, mood changes, the need for stimulation and craving, and Fear of missing out (FOMO) (Billieux et al., 2015; Anghelcev et al., 2020; Fernandez et al., 2020). Emotional reactions are also associated with distress when binge-watching is blocked. It is possible that in the case of compulsive viewing of series, similar symptoms of abstinence occur as in the case of other behavioral addictions. However, in order to confirm this relationship, further research on the problematic binge-watching is undoubtedly needed.

Additionally, research has shown that the anxiety-depressive syndrome is associated with a greater frequency of loss of control over binge-watching behaviors and neglect of duties. At this point, it is worth mentioning that problems with self-control and neglect of duties are characteristic of a low level of conscientiousness, which in turn is characteristic of individuals exhibiting excessive binge-watching behavior (Govaert, 2014; Chambliss et al., 2017; Tóth-Király et al., 2017; Anghelcev et al., 2020). An individual escapes from negative emotional states by watching TV series. This behaviour can be both a distraction and a source of the positive gratification. Consequently, the viewer can lose control over the amount of time spent on binge-watching, and as a result they can neglect their duties related to work, school, or home, which may lead to the release of further negative emotions. A vicious circle mechanism characteristic of addictions is created (Woronowicz, 2009; Grzegorzewska and Cierpiałkowska, 2018; Cierpiałkowska and Chodkiewicz, 2020). Furthermore, research by Panda and Pandey (2017) indicates that if an individual experiences guilt or fear after the end of a binge-watching session, they are more likely to quickly re-engage in binge-watching behaviors in order to temporarily avoid negative feelings. Researchers indicate that as a result of this phenomenon, individuals may become increasingly dependent on binge-watching. At this point, it should also be mentioned that the results of the research indicated that the anxiety-depressive syndrome is an important predictor for the manifestation of escape motivation – an individual watches series in order to escape from everyday problems and negative emotions. The literature on the subject describes research that emphasizes the importance of escape motivation for displaying excessive binge-watching behaviors (Panda and Pandey, 2017; Castro et al., 2019; Flayelle et al., 2019b; Ort et al., 2021). Interestingly, the results of the research showed no significant effect of the anxiety-depressive syndrome on the entertainment, social, and informative motivation. Perhaps, seeking entertainment is not significant for the people with anxiety-depressive syndrome. However, it is important to mention that the entertainment motivation has direct positive effect on the symptoms of problematic binge-watching. Research in the literature shows that the entertainment motivation is the most frequently indicated motivation among binge-watchers (Pittman and Sheehan, 2015; Panda and Pandey, 2017; Rubenking and Bracken, 2018; Castro et al., 2019; Flayelle et al., 2020; Starosta and Izydorczyk, 2020). People binge-watch because they want to relax and be entertained. Furthermore, it is important to mention that social motivation is the only type of motivation to watch TV series which have no effect on problematic binge-watching. The anxiety-depressive syndrome also had no predictive value for social motivation. The lack of interactions between those variables and significance of motivation to deal with loneliness could be explained by the occurrence of interpersonal problems characteristic for people with high intensity of anxiety and depression (Butcher et al., 2020). It can be assumed that creating parasocial relationships with fictional character may be easier and less threatening than engaging in real social interactions (Wheeler, 2015; Bernhold and Metzger, 2020).

The anxiety-depressive syndrome is a strong predictor of problematic binge-watching symptoms, such as negative health consequences, preoccupation, and negative social consequences. So far, not many studies have been carried out on the impact of binge-watching on the health of individuals, but few studies in the literature have shown that binge-watching is associated with worse sleep quality and unhealthy, and irregular diet (Exelmans and Van den Bulck, 2017; Vaterlaus et al., 2019; Anghelcev et al., 2020; Dixit et al., 2020). On the other hand, research indicates that individuals prefer to binge-watch alone so the quantity of time they spend watching TV series may affect their interpersonal relationships by further reducing the number of contacts with other people (Wheeler, 2015; Sun and Chang, 2021). At this point, it is worth mentioning that depressive and anxious people often feel lonely and often may not be motivated to keep in touch with other people (Butcher et al., 2020). The lack of energy necessary for social interactions combined with a simultaneous sense of social isolation explains such a strong positive relation between the anxiety-depressive syndrome and the motivation to cope with loneliness caused by watching TV series and the negative relation with social motivation. The characters of the series replace social connections so that individuals do not feel so lonely (Starosta et al., 2019; Bernhold and Metzger, 2020; Ort et al., 2021). The results gathered by Erickson et al. (2019) highlight that people who binge-watch more are characterized by creating stronger parasocial bonds with the characters of the series. Moreover, research by Rosaen and Dibble (2015) points out that the desire to belong and attachment anxiety are predictors of parasocial relation formation.

It should be mentioned that the research results presented an indirect influence of the motivation to deal with loneliness and spending free time on the frequency of sessions within 1 month. Perhaps, individuals with such motivations are characterized by a greater frequency of sessions undertaken, because they do it out of habit and boredom, or they treat the series as a companion when they feel lonely. The literature on the subject confirms the existence of such dependencies (Pittman and Sheehan, 2015; Flayelle et al., 2020). Research by Sung et al. (2018) indicates that people who watch series to “pass the time” more often show higher levels of binge-watching.

Interestingly, informative motivation has a direct impact on most of the problematic binge-watching symptoms, in addition to negative health consequences. Informative motivation is related to the desire to meet the cognitive needs of an individual by watching TV series. The greatest impact of informative motivation surfaces in case of preoccupation and emotional reactions, which is understandable because this scale describes the emotional and cognitive involvement of an individual in binge-watching as well as looking for additional information about TV series. Perhaps, such a strong role of informative motivation is associated with FOMO – fear of missing out, the role of which for binge-watching behaviors is highlighted by Conlin et al. (2016) in their research. These authors assume that binge-watchers with high intensity of fear of missing out want to collect information that will enable them to participate in social discourse and prevent them from being ostracized in conversation with others (Conlin et al., 2016). However, research conducted by the authors of this article did not confirm the role of the social motivation mentioned by Conlin et al. (2016). Maybe problematic binge-watchers focus on cognitive and not on social aspects of this behavior. Interestingly, people who binge-watch more, the so-called “heavy binge-watchers,” experience FOMO more frequently (Anghelcev et al., 2020). Another explanation for the role of informative motivation is the use of the immersive function of binge-watching as a behavior that strongly engages the cognitive and emotional processes of an individual, which may be associated with a sense of being lost in a fictional world (Conlin, 2015; Petersen, 2016; Walter, 2018, Erickson et al., 2019). It is worth mentioning that the informative motivation influences lying and, to a lesser but still significant extent, negative social consequences and emotional reactions. It is possible that an individual who desires cognitive impressions involved cognitively in binge-watching has a greater tendency to hide the truth about the amount of time spent on binge-watching.

Another interesting finding of this research is that motivation to watch TV series has weak mediating effect between the anxiety-depressive syndrome and the symptoms of problematic binge-watching. Anxiety depressive syndrome has significant and weak indirect effect through motivation to deal with loneliness, motivation to spend free time, escape motivation on symptoms of problematic binge-watching, and frequency of binge-watching sessions. The direct effects of independent variable were strong and medium. It seems that motivation can weaken the effects of anxiety-depressive syndrome. Due to the fact that motivation to watch TV series has also direct effect on the problematic binge-watching, it can be assumed that they may have more predictive than mediating value for explaining binge-watching. However, it requires further research.

### Limitations of the Study

An unquestionable limitation of the study was applying the stratified sampling method of the research group. As a result, we are not able to generalize the collected results to the entire population. They mainly concern young people between the ages of 18 and 30. In the future, it will be necessary to conduct research on other age groups in order to check whether the discussed dependencies will also be characteristic of adolescents, middle-aged people, and seniors. Another limitation of the study was the predominance of women (*n* = 537) compared to men (*n* = 98). It is very likely that the underlying reason is conducting the study among students mainly of humanities, where more women than men study. According to the data of the Central Statistical Office (2019), (GUS, 2020) and the report of the Ministry of Education and Science (2020), Polish women not only study particular disciplines more often, but also more often decide to undertake higher education. The predominance of women in the research group could also be caused by the greater willingness of women to participate in the research (Smyth, 2008; Lobato et al., 2014). In addition, the results of systematic review show that women more often took part in research on binge-watching than men (Flayelle et al., 2020; Starosta and Izydorczyk, 2020). Perhaps, binge-watching itself is a more interesting research topic for women than for men, which is why they take part in research on this phenomenon more often. In the future, it will undoubtedly be valuable to conduct studies on a larger population of men in order to confirm the results obtained in the discussed study. Another research limitation was conducting it during the global COVID-19 pandemic, which undoubtedly influenced the emotional state of the subjects and their activities. As a result of the lockdown, the activity of the respondents was limited to tasks and entertainment undertaken at home. It is therefore possible that as a result of the lack of alternatives, people binge-watched more often than they would have done in other circumstances. Research by Dixit et al. (2020) has shown that people binge-watched more during the pandemic than before. The popularity of the given activity during the pandemic may also be proved by statistics, such as the subscribers increase of one of the streaming platforms. Netflix had 183 million subscribers worldwide in the first quarter of 2020, while that number went up to 204 million in the fourth quarter (Jay, 2021). The results of the study showed that the respondents were binge-watching out of boredom and because they felt lonely (Dixit et al., 2020). Binge-watching has become a way of coping with stress, which was supported by the high availability of streaming services and granting immediate gratification. The authors of the research indicate that resorting to binge-watching in situations of emotional distress may not disappear with the end of the pandemic, indicating the existence of a developing risk of behavioral addiction to binge-watching (Dixit et al., 2020; Kar et al., 2020). Due to the undoubted impact of the pandemic on the frequency of binge-watching and the motivation to watch TV series, it will be necessary to conduct comparative studies after the end of the pandemic in order to check whether the drawn conclusions are still valid in the post-pandemic situation.

### Implications

Studies imply that binge-watching can be both entertaining and potentially addictive behavior (Flayelle et al., 2020; Starosta and Izydorczyk, 2020). This research shows that such personal factors as anxiety-depressive syndrome and specific motivations to watch TV series are important conditions of problematic binge-watching. Such factors may hinder the healthy and harmonious engagement in binge-watching. Moreover, it is important to mention that problematic binge-watching can also harmfully affect other health-related behaviors, such as healthy diet, sleep, and physical activity (Exelmans and Van den Bulck, 2017; Vaterlaus et al., 2019; Anghelcev et al., 2020; Dixit et al., 2020). Consequently, engaging in problematic binge-watching may inhibit the healthy lifestyle changes. The occurrence of problematic binge-watching implies the need of creating some preventive and therapeutic interventions. However, health-related changes in relation of problematic binge-watching may encounter some obstacles. Firstly, binge-watching usually is not perceived as socially harmful behavior (Grzegorzewska and Cierpiałkowska, 2018; Ort et al., 2021). Secondly, the accessibility of new technologies and some structural factors of streaming platforms are made to keep viewer’s engagement which can lead to loss of the control (Alter, 2017; Flayelle et al., 2020). Thirdly, there is still inconsistency in defining “normal” and problematic binge-watching (Flayelle et al., 2020; Starosta and Izydorczyk, 2020). Due to that, gaining further knowledge about psychological conditions of problematic binge-watching may be important for the future diagnostic, preventive, and therapeutic implementations.

### Conclusion

In recent years, binge-watching has become one of the most popular forms of pastime among the young generation. Research shows that binge-watching can be both a typical hobby and a possible anti-health behavior, with some similarities to other behavioral disorders. Developing easily accessible streaming platforms and new ways of creating narrative of TV series changed the way people consume the media. Such factors and the individual psychological predispositions could change the harmless and entertaining way of spending free time, and contribute to the development of problematic viewing. The results of this study showed that the anxiety-depressive syndrome is a strong predictor of the problematic binge-watching symptoms. Thus, it influences the activities undertaken by an individual. In addition, anxiety-depressive syndrome is also a strong predictor of specific motivations to watch TV series. The anxiety-depressive syndrome correlates positively with the escape motivation, the motivation to deal with loneliness, and the motivation to spend free time. These motivations mediate between the anxiety-depressive syndrome, syndrome of problematic binge-watching, and frequency of binge-watching session during 1 month. Informative and entertainment motivations directly influence the symptoms of problematic binge-watching. There is no effect of social motivation on the symptoms of problematic binge-watching. Motivation to cope with loneliness and the motivation to spend free time indirectly influence the frequency of binge-watch sessions during 1 month. The research results contributed to the increase of knowledge about the psychological determinants of binge-watching. Excessive binge-watching behaviors can be used as an element of affective self-regulation and may also condition the functioning of an individual in society and carrying out one’s duties. Nevertheless, further studies on various populations are necessary to confirm the obtained results. Further research on the phenomenon and delineation of the boundaries between healthy and harmonious binge-watching, and unhealthy-problematic binge-watching is important both in order to take possible preventive and therapeutic measures and to avoid excessive degeneration of everyday life.

## Data Availability Statement

The raw data supporting the conclusions of this article will be made available by the authors, without undue reservation.

## Ethics Statement

The studies involving human participants were reviewed and approved by the Institute of Applied Psychology, Faculty of Management and Social Communication, Jagiellonian University, Cracow, Poland. The participants provided their written informed consent to participate in this study.

## Author Contributions

JS: research idea, research design, conceptualization, literature review, data collection, data interpretation, and draft manuscript. BI: research design, conceptualization, work supervision, and revision of work. AW: data collection and data interpretation. All authors contributed to the article and approved the submitted version.

### Conflict of Interest

The authors declare that the research was conducted in the absence of any commercial or financial relationships that could be construed as a potential conflict of interest.
